# Insights into the Membrane Interactions of the Saposin-Like Proteins *Na*-SLP-1 and *Ac*-SLP-1 from Human and Dog Hookworm

**DOI:** 10.1371/journal.pone.0025369

**Published:** 2011-10-03

**Authors:** Charlene Willis, Conan K. Wang, Asiah Osman, Anne Simon, Darren Pickering, Jason Mulvenna, Alan Riboldi-Tunicliffe, Malcolm K. Jones, Alex Loukas, Andreas Hofmann

**Affiliations:** 1 Parasite Cell Biology, Queensland Institute of Medical Research, Herston, Queensland, Australia; 2 Structural Chemistry Program, Eskitis Institute for Cell and Molecular Therapies, Griffith University, Nathan, Queensland, Australia; 3 Université Lyon 1, CNRS, ICBMS, UMR 5246, F-69622 Villeurbanne, France; 4 James Cook University, Cairns, Queensland, Australia; 5 Australian Synchrotron, Clayton, Victoria, Australia; 6 School of Veterinary Sciences, The University of Queensland, Gatton, Queensland, Australia; 7 Department of Veterinary Science, The University of Melbourne, Victoria, Australia; 8 Queensland Tropical Health Alliance, Townsville, Queensland, Australia; University of Queensland, Australia

## Abstract

Saposin-like proteins (SAPLIPs) from soil-transmitted helminths play pivotal roles in host-pathogen interactions and have a high potential as targets for vaccination against parasitic diseases. We have identified two non-orthologous SAPLIPs from human and dog hookworm, *Na*-SLP-1 and *Ac*-SLP-1, and solved their three-dimensional crystal structures. Both proteins share the property of membrane binding as monitored by liposome co-pelleting assays and monolayer adsorption. Neither SAPLIP displayed any significant haemolytic or bactericidal activity. Based on the structural information, as well as the results from monolayer adsorption, we propose models of membrane interactions for both SAPLIPs. Initial membrane contact of the monomeric *Na*-SLP-1 is most likely by electrostatic interactions between the membrane surface and a prominent basic surface patch. In case of the dimeric *Ac*-SLP-1, membrane interactions are most likely initiated by a unique tryptophan residue that has previously been implicated in membrane interactions in other SAPLIPs.

## Introduction

The human hookworms, *Ancylostoma duodenale* and *Necator americanus* (Nematoda, Ancylostomatoidea), infect more than half a billion people worldwide [1], and are agents of severe morbidity in affected humans from many developing nations [2]. Hookworms are voracious and wasteful feeders of blood, and the cumulative effects of their feeding activity leads to chronic disease, notably iron deficiency anaemia [3]. Vaccine strategies for hookworm diseases are focused on reducing morbidity by targeting haemolytic capacity of the hookworm digestive system. In this regard, apical molecules of the hemoglobinolytic cascade are prime targets for immunoprophylaxis of hookworm disease.

Among the proteins with potential for development as anti-parasite vaccines are the family of saposin-like proteins (SAPLIPs). SAPLIPs from the liver flukes *Fasciola hepatica* and *Clonorchis sinensis*, and the protistan *Entamoeba histolytica* have noted haemolytic capacity [4], [5] and are likely to play key roles in initiating host cell digestion. A recombinant *F. hepatica* SAPLIP, *Fh*-SAP-2, showed protective efficacy against challenge infection with *Fasciola hepatica* [4], and provided cross-protection in mice experimentally infected with the highly pathogenic human blood fluke, *Schistosoma mansoni* [6]. These results indicate that SAPLIPs play important roles in parasite homeostasis in their hosts and have potential as attractive broad-band protective targets for vaccinations.

Saposins of mammals are activators of sphingolipid hydrolases, and their fold is the characteristic feature of the large and diverse family of SAPLIPs. Human saposins are produced in late endosomes or lysosomes by proteolysis of prosaposin into four small proteins, called SapA-SapD. A primary role for saposins is to load hydrophobic lipid antigen from lysosomal membranes onto human CD1 molecules for further processing by the immune system [7]. Saposins are also involved in degrading glycosphingolipids and promote their hydrolytic processing by exohydrolases [8]. Despite their similar structure, each saposin targets a distinct sphingolipid and enables its degradation by a partially overlapping set of enzymes [9], [10]. In line with these activities, saposins possess membrane binding and lipid transport properties, and they have been shown to destabilise phospholipid vesicles, modulating the membrane structure in a detergent-like manner [11].

The conserved core structure of SAPLIPs, the Sap domain, has the topology of a four- or five-helix bundle with six conserved cysteine residues that are involved in forming the characteristic disulphide bond pattern [12]. This fold is adapted to carry out a number of different functions at biological membranes. Accordingly, despite possessing the same fold, individual SAPLIPs generally share little amino acid sequence identity. SAPLIPs or Sap domains have been identified in proteins with diverse functions, ranging from cytolytic proteins from amoebae [13], granulysin [14], NK-lysin from pig [15], jellyfish lens crystallin [16], plant aspartic protease [17] to neurotrophic factors [18].

Previous structural studies have shown that saposins have an exceptional level of conformational flexibility. At least two configurations have been observed for saposins: the substrate-free closed form and the ligand-bound open form. In the absence of lipids or detergents, saposins adopt a compact four-helix bundle fold, with the hydrophobic lipid binding site hidden in the protein core. In the presence of lipids or SDS, the helix bundle of SapB [19] and SapC [20] opens to form a V-shaped configuration, exposing the ligand binding site. It is believed that the opening and closing of the saposin fold is fundamental to their mechanism of action [21].

Although the exact mode of enzymatic activation by saposins is still unknown, it is generally believed that these proteins remodel the membrane, and directly interact with lipid degrading enzymes, thus providing a platform for lipid hydrolases [22]. As with saposins, SAPLIPs have varied functions [23] most of which appear to involve an interaction with lipids. Based on structural knowledge available for amoebapore A from *Entamoeba histolytica*, it is assumed that dimerisation is regulated by protonation of an easily accessible histidine residue at low pH [13]. The assembly of three dimers is then thought to constitute the pore forming species through the lipid bilayer of the target cell [13].

Membrane extracts of the canine hookworm *Ancylostoma caninum*, an avid blood feeder, have been shown to be haemolytic [24], an action arising from molecular complexes that form pores in erythrocyte membranes. Our transcriptomic analyses of human and canine hookworm digestive tissues [25] uncovered novel SAPLIPs, which were partially characterised in a recent study [26]. However, lytic activity of the recombinant *Ac*-SLP-1 from *A. caninum* was not observed, and it was thought that this was due to the presence of a fused purification tag in the recombinant protein which may have interfered with the oligomerisation of the protein.

In this study, we investigate the structure and biochemical functions of two SAPLIPs from human (*Necator americanus*) and dog hookworm (*Ancylostoma caninum*), *Na*-SLP-1 and *Ac*-SLP-1. Both SAPLIPs were identified from EST datasets, expressed and purified as recombinant protein in its mature form. In order to gain insights into their structure-function relationships and possible roles in host-pathogen interactions, we determined the three-dimensional crystal structures and investigated their biochemical properties as well as functional implications.

## Results

### Identification and production of recombinant *Na*-SLP-1 and *Ac*-SLP-1

By database search, a single *N. americanus* EST sequence (gi|22528669) was identified that contained an autonomous SAPLIP domain. This sequence coded for a full length protein with an ORF of 137 amino acids, including a signal peptide of 20 amino acids. Being the first SAPLIP reported from *N. americanus*, we have denoted this protein as *Na*-SLP-1.

Recombinant *Na*-SLP-1 and *Ac*-SLP-1 were expressed in *P. pastoris* and purified as soluble proteins. In bacterial expression, the proteins were only obtained in inclusion bodies. Using a homologous mouse antiserum (see Materials and Methods S1), proteins expressed in yeast were detected by Western blot, and their identity further verified using limited proteolysis and mass spectrometry. Amino acid sequences belonging to *Na*-SLP-1 and Ac-SLP-1 were unambiguously identified from the MS fingerprinting. Mass spectrometry of *Na*-SLP-1 indicates that the protein degrades over time, whereas *Ac*-SLP-1 remains intact (9531 g/mol; theoretical M = 9531 g/mol).

### Solution structure

**Figure 1:**
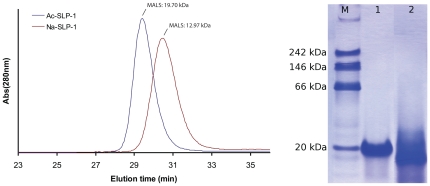
Quaternary structure in solution. ***Left***: Size exclusion with MALS detection reveals monomeric *Na*-SLP-1 and dimeric *Ac*-SLP-1 at pH 7.5 and 5.0 under normal salt conditions (representative chromatograms shown). ***Right***: *Ac*-SLP-1 is exclusively observed as a dimer on native gels. The protein migrates at 20 kDa under native conditions (Lane 1), but at smaller sizes in the presence of SDS (Lane 2). The migration behaviour of *Na*-SLP-1 on native gels does not allow analysis due to smeared bands (data not shown).

With size exclusion chromatography (see Figure 1), the quaternary structure of the two hookworm SAPLIPs was assessed at neutral (pH 7.5) and acidic pH (pH 5.0). *Ac*-SLP-1 exists exclusively as a dimer with an experimentally determined molecular mass of 19700 g/mol (MALS; theoretical M = 19062 g/mol).

*Na*-SLP-1, in contrast, is exclusively monomeric (MALS 12970 g/mol; theoretical M = 13530 g/mol). For *Ac*-SLP-1, these results agree with the migration behaviour on native gels where a clear dimer band is observed.

### Crystal structures

**Figure 2:**
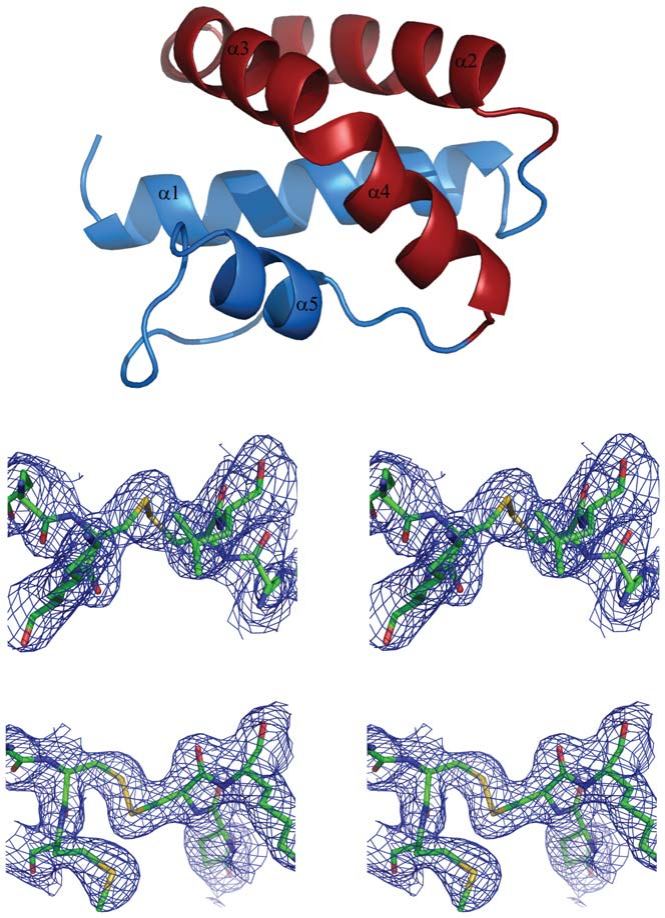
Both SAPLIPS adopt the fold of the Sap domain. ***Top***: Cartoon representation of the crystal structure of *Na*-SLP-1 with the colour scheme highlighting the jaw-like arrangement of the five α-helices. ***Middle***: Stereo figure of the 2F_o_-F_c_ density around the Cys-39 - Cys-49 disulphide bond of *Na*-SLP-1. ***Bottom***: Stereo figure of the 2F_o_-F_c_ density around the corresponding region in *Ac*-SLP-1 (Cys-42 - Cys-56). The electron density is contoured at 1σ. Figure prepared with PyMOL [48].

The structure of both hookworm SAPLIPs were determined in hexagonal space groups, and closely resemble the fold of the Sap domain, as known from saposins and other SAPLIPs. The fold is comprised of a four- or five-helix bundle, where a kink in the third helix can lead to occurrence of two helices, α3 and α4 (see Figure 2). For the purpose of this paper, the C-terminal helix will be denoted as α5 throughout. The terminal helices α1 and α5 on the one hand, and helices α2, α3 and α4 on the other, form a jaw-like arrangement. Three conserved dithioether bonds link helix α1 with the C-terminal end, helices α1 and α5, and helices α2 and α3.

In the crystal structure of *Na*-SLP-1, the C-terminal extension of about 30 amino acids, which is attached to the N-terminal Sap domain could not be resolved, which agrees with the results from mass spectrometry. The crystal structure contained two molecules in the asymmetric unit, giving rise to a complex network of molecules in the crystal packing with a variety of different protein-protein contacts. The two monomers in the asymmetric unit agree very well in their overall fold as is reflected by the Cα rms deviation of 0.55 Å. The protein surface built by helices α2 and α3 shows a clustering of seven basic residues, namely Arg-28, Lys-36, Lys-40 (α2) and Arg-46, Arg-47, Arg-48 and Lys-54 (α3). This basic surface patch may provide a binding interface to the head groups of a phospholipid membrane.

**Figure 3:**
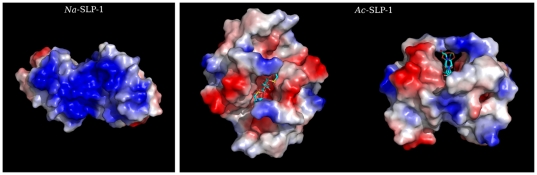
The crystal structures suggest possible dimers for both hookworm SAPLIPs. ***Left panel***: Crystal packing analysis reveals a P2-symmetric dimer for *Na*-SLP-1 (571 Å^2^). Note the extended basic surface patch observed in the P2-symmetric dimer on the left. ***Right panel***: Top-on (left) and face-on (right) views of the crystallographic dimer of *Ac*-SLP-1. A citrate molecule flanked by two HEPES molecules is bound in the acidic groove in a special position. Figure prepared with PyMOL [48].

Despite crystallising in the same crystal system and similar cell dimensions as SLP-1 from human hookworm, the crystal structure of *Ac*-SLP-1 possesses only one molecule in the asymmetric unit. Consequently, large solvent channels and a lesser variety of protein-protein contacts are observed in this structure. There was no electron density observed for the very N-terminal residues (amino acids 1–6), most likely because of high flexibility of this region (see also Figure S1). Further support for this assumption comes from the fact that the visible part of the N-terminal region projects into a solvent channel and thus lacks the possibility of forming interactions with side chain residues. Near the N-terminal region, a citrate molecule was found to occupy a special position on a two-fold axis (see Figure 3). Parallel to the citrate, a large oblate difference density with an apical branch towards the citrate is observed. Since this density clearly is isolated from the continuous protein density, it appears to belong to another solvent molecule. Shape and extent suggested that this density may be fitted with a HEPES molecule, albeit the atomic displacement parameters (B-factors) remain rather high after refinement. Overall, the B-factors in this structure are elevated, with very high values observed in the linker region between helices α1 and α2. In contrast to the human hookworm SLP-1, *Ac*-SLP-1 does not possess a prominent cluster of basic surface residues, but rather three small isolated basic surface areas, comprised of Lys-17/Lys-21 (α1), Lys-36 (α2) and Lys-54/Lys-57 (α3). Two of those basic areas are located on the surface built by helices α1 and α3 which otherwise appears rather hydrophobic and extremely flat. Notably, in the crystal, this surface is in intimate contact with a symmetry-related molecule, thus establishing a P2-symmetric crystallographic dimer (Figure 3). A large hydrophilic cavity with a volume of 177 Å^3^ is lined by residues from the very N-terminal region (α1) and residues located on α2 and α3. The cavity is situated at one side of the *Ac*-SLP-1 molecule that allows access to the linear groove and eventually the flat hydrophobic surface on α3.

### Structural comparison with other Sap domain containing proteins

Comparison of the crystal structure of these hookworm SLP-1s with structures of saposins and other SAPLIPs highlights the intrinsic flexibility within the fold of the Sap domain. The backbone rms deviations compared to other Sap domains (see Table 1) are larger than 1.9 Å (*Na*-SLP-1) and 2.2 Å (*Ac*-SLP-1). This flexibility is not only due to the different side chain interactions and subtle changes in the positioning of the secondary structure elements. Within the framework of the same underlying backbone fold, there is also variation as to the macromolecular shape of the individual proteins. This is best illustrated by the α3 helix, which in *Ac*-SLP-1 builds a flat surface, whereas the corresponding helix of *Na*-SLP-1 contributes to a much more globular shape of the molecule. Additionally, as pointed out by Eisenberg and colleagues for granulysin [14], the loose packing of side chains is observed with many Sap domains. This packing results in large void volumes in the core, thus making the core oily. For *Na*-SLP-1, we found 14 void volumes with values larger than 5 Å^3^, the largest ones being 29 Å^3^, 30 Å^3^ and 43 Å^3^, using the same probe radius as in the granulysin study [14] (see Materials and Methods S1). *Ac*-SLP-1, in contrast, shows no such feature and has a well ordered internal side chain packing. No void volumes were detected using the same protocol as for *Na*-SLP-1. However, helix α2, and especially the loop linking α2 and α1, shows very high B-factors and is thus a flexible part of the molecule.

**Table 1:** Structural superposition of hookworm SLP-1 proteins with known saposin and SAPLIPs.

			*Na*-SLP-1	*Ac*-SLP-1
	Conformation of Sap fold	PDB accession code	rms deviation (Å)	rms deviation (Å)
*Na*-SLP-1 (mol A)	closed		-	3.2
*Na*-SLP-1 (mol B)	closed		0.55	3.1
pore forming protein from *E. histolytica*	closed	1of9	2.3	3.1
caenopore-5 (slp 5 from *C. elegans*)	closed	2js9	2.6	3.2
caenopore-5 (slp 5 from *C. elegans*)	closed	2jsa	2.5	2.3
human granulysin	closed	1l9l	2.5	2.5
human saposin B	open	1n69	2.0	2.9
human saposin C	closed	1m12	2.2	2.3
human saposin C	closed	2gtg	2.1	2.2
human saposin C	open	2qyp	2.8	2.6
human saposin C	open	2z9a	2.0	3.0
human saposin C in SDS micelles	open	1sn6	3.1	2.8
human saposin D	closed	2rb3	1.9	2.6

Superposition calculations were carried out using the SSM algorithm implemented in SUPERPOSE in the CCP4 suite [42].

### Oligomeric structure in the crystal

Crystal packing analysis of *Na*-SLP-1 reveals a P2-symmetric dimer, albeit with a moderate interface area (571 Å^2^). Despite being a monomeric protein in solution, this dimer may possess a functional importance in the membrane-bound state, since it combines the basic surface clusters of the two monomers into one large patch (Figure 3).

*Ac*-SLP-1, despite being a monomer in the asymmetric unit, crystallised as a P2-symmetric dimer that is aligned with one of the crystallographic axes. Buried within the dimer interface is a flat hydrophobic surface constituted mainly by the solvent-exposed side of helix α3. The buried surface area of 767 Å^2^ corresponds to about 30% of the monomer surface, and is thus above the threshold for specific interaction interfaces as established by Janin [27]. The most striking feature of the *Ac*-SLP-1 dimer is a long linear groove connecting the large cavities at their distal ends. The groove exhibits an electronegative surface potential throughout and two clamps at the solvent-exposed side which are provided by the N-terminal asparagine residues.

### Membrane interactions

**Figure 4:**
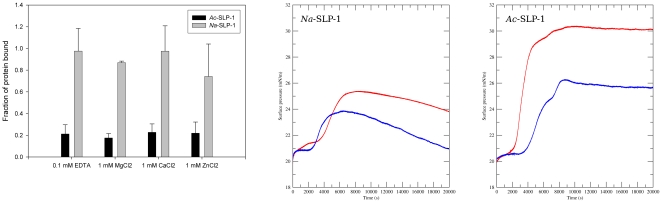
Experimental membrane interactions. ***Left panel***: Liposome co-pelleting assays using multilamellar vesicles (DOPS/DOPE at molar ratio of 3∶1) at neutral pH shows binding of both SAPLIPs independent of metal ions. While for *Na*-SLP-1 full binding is observed, *Ac*-SLP-1 binding levels are ∼20%. ***Middle/right panels***: Adsorption of both SAPLIPs to monolayers (DOPS/DOPE at molar ratio of 1∶3) was assessed in a Langmuir surface film balance at neutral (pH 7.5, blue curves) and acidic conditions (pH 4.7, red curves). While quantitatively different, three phases are observed for both proteins under all tested conditions: an immediate-onset Langmuir-like adsorption with small surface-pressure increase (phase 1, peripheral binding), followed by a large surface pressure increase (phase 2, embedding into the membrane), and a final stage with either stable (*Ac*-SLP-1) or decreasing (*Na*-SLP-1) surface pressure (phase 3).

Membrane binding of both SAPLIPs was first assessed in a liposome co-pelleting assay using MLVs composed of DOPS/DOPC (3∶1) in the absence and presence of several divalent metal ions at pH 7.5. Consistently, a rather low binding (∼20%) was observed for *Ac*-SLP-1; in contrast, *Na*-SLP-1 bound quantitatively to liposomes. Membrane binding of both SAPLIPs is independent of divalent metal ions (Figure 4).

Evaluation of membrane binding by surface film balance measurements conducted under area control confirms the qualitative difference in membrane interactions between the two SAPLIPs but also revealed common additional features. A membrane composition with less content of phosphatidylserine (DOPS/DOPE at a molar ratio of 1∶3) was chosen deliberately to minimise the effects of non-specific electrostatic interactions. For both proteins, three stages of monolayer adsorption were observed (Figure 4). Following injection of the proteins into the subphase, a Langmuir-like adsorption with immediate on-set and small change in surface pressure (Δπ = 0.5–0.8 mN m^−2^) is evident (phase 1). This is followed by a steep increase of surface pressure (Δπ = 3–9 mN m^−2^) with sigmoid shape, indicating the presence of a cooperative process (phase 2). After ∼1 hour, this process is completed and the membrane surface pressure remains stable at the high level in case of *Ac*-SLP-1 for several hours (phase 3). For *Na*-SLP-1, a slow decline in surface pressure is registered and the value of π eventually arrives at levels similar to π _0_.

Comparing the monolayer surface adsorption at neutral and acidic pH, two main differences became apparent. Firstly, at acidic pH, phase 2 starts earlier than under neutral conditions and its onset happens during the initial Langmuir-type adsorption. Secondly, the surface pressure increase during phase 2 is more pronounced at acidic pH (Δπ = 4.5 or 9 mN m^−2^ for *Na*-SLP-1 or *Ac*-SLP-1, respectively) than under neutral conditions (Δπ = 3 or 5 mN m^−2^).

The qualitative differences in monolayer surface adsorption between both SAPLIPs are apparent and consistent at neutral and acidic pH. For *Na*-SLP-1, the initial Langmuir-like adsorption phase is more pronounced as reflected by the larger surface pressure difference when compared to *Ac*-SLP-1. Also, both SAPLIPs differ in their behaviour in phase 3, where *Na*-SLP-1 shows a significantly smaller surface pressure difference as well as a subsequent loss of surface pressure. This is contrasted by higher surface pressure differences maintained for a very long time in the case of *Ac*-SLP-1.

The ability of both hookworm SAPLIPs to disintegrate membranes *in vitro* was evaluated using a liposome stability assay. Here, a fluorescent dye is encapsulated into lipid vesicles at self-quenching concentrations. Leakage of dye through the membrane is detected by an increase in the fluorescence signal. Neither hookworm SAPLIP elicited a detectable efflux of fluorescence dye (data not shown), which is in qualitative agreement with the stable surface pressure seen in phase 3 of the monolayer adsorption experiments that indicates a stable monolayer.

### Biological activity

To assess the biological function of these proteins, biologically relevant functional assays were carried out to assess haemolytic and bactericidal activity (see Materials and Methods S1). A co-pelleting assay using red blood cells to determine binding to the cell membranes showed unselective binding of both hookworm proteins. All control proteins (Scabies Mite Inactive Protease Paralogues, *S. mansoni* Tetraspanin 2) also bound to the red blood cells (data not shown), presumably due the high negative surface charge. Neither *Na*-SLP-1 nor *Ac*-SLP-1 possessed any detectable haemolytic activity when assessed at either pH 5.0 or pH 7.0, in the presence or absence of divalent cations. This agrees with the findings from the liposome stability assay where no detectable lytic activity was observed (data not shown). Bactericidal assays did not show any inhibition of growth of *E. coli* by either *Ac*-SLP-1 or *Na*-SLP-1 (data not shown).

## Discussion

Parasite SAPLIPs share low sequence identity with human saposins (11–27% amino acid sequence identity between the hookworm SAPLIPs and human saposins B, C and D). We have solved the first three-dimensional crystal structures of SAPLIPs from hookworms and from ecdysozoan organisms, namely *Na*-SLP-1 and *Ac*-SLP-1. The two distinct proteins are not orthologues which is clearly apparent from their structural features. However, both SAPLIPs share the property of membrane binding and the absence of any detectable haemolytic activity.

The mechanisms by which members of the family of proteins containing Sap domains contact with membranes is variable. An insertion of the N- and C-termini into the membrane has been shown for human saposins A and C [28]. Similar contacts are proposed for NK-lysin and have led to the suggestion that this protein uses the molecular mechanism of electroporation to destabilise the membrane [29]. Granulysin, in contrast, appears to make contact with membranes through a cluster of exposed basic residues. Eisenberg and colleagues [14] proposed a model where further positive surface charges located off the initial membrane binding patch may then be brought closer to the membrane by a rolling mechanism. This may be achieved by sliding of the two lobes of granulysin (α1/α5, α2/α3/α4) against each other, resulting in a scissor-like motion. The scissor opening would expose a more lytic surface of the protein (the most lytically active peptides reside in the inner part of this protein [30], [31]), and also drill the protein into the membrane, thereby bending or tearing it.

### Proposed membrane binding mechanisms of *Na*-SLP-1

**Figure 5:**
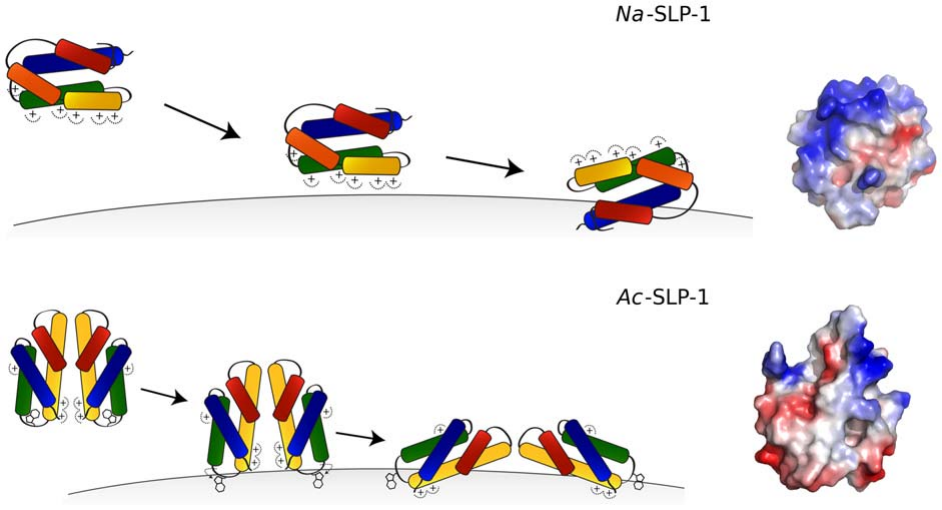
Potential membrane binding mechanisms of *Na*-SLP-1 and *Ac*-SLP-1. ***Top***: *Na*-SLP-1 is expected to make contact with the membrane surface through electrostatic interactions. Once at the membrane, the protein can roll the surface of helix α1 (see view on the right) onto the membrane which may be embedded into the membrane interior. ***Bottom***: Initial contact of the *Ac*-SLP-1 dimer with the membrane may be through Trp-48 in a swing-out conformation. This may cause the dimer to disassemble and expose the rather hydrophobic dimer interface (pictured on the right) which will be embedded into the membrane.

The crystal structure of *Na*-SLP-1 reveals the presence of seven exposed basic residues on helices α2 and α3, giving rise to an extensive basic patch even on one monomer (see Figure 3) which is likely to initiate contact with the membrane surface, driven by electrostatic interactions (Figure 5). The peripheral adsorption to the membrane surface is readily apparent from the Langmuir-like behaviour of the monolayer surface adsorption experiments (phase 1). The subsequent large increase in surface pressure suggests that the protein embeds or inserts itself into the membrane. The activity is likely to require rotation of the protein with respect to the membrane surface in order to bring the hydrophobic surface areas on *Na*-SLP-1 (located on helix α1) into contact with the hydrophobic interior of the membrane.

This process may be hampered to some extent by the presence of negatively charged side chains (e.g. Asp-8, Glu-38) on this face of the protein which is reflected by the earlier onset of phase 2 and the larger surface pressure and the difference between phase 1 and 3 in the monolayer adsorption experiments conducted at acidic pH.

Such a scenario bears resemblance to the mechanism proposed for granulysin [14] which is also thought to make initial contact with the membrane by electrostatic interactions, albeit not through a prominent basic surface patch. Intriguingly, *Na*-SLP-1 and granulysin share the feature of a loosely packed protein core, as evident from void volumes in the interior of both proteins. Based on the current crystal structure, calculation of void volumes resulted in significantly larger values for *Na*-SLP-1 (14 compartments with values of 5–42 Å^3^ located in the interface between the upper and lower “jaws”) than granulysin (5–14 Å^3^) or the plant saposin prophythepsin (15 Å^3^) [14].

It is thus surprising that *Na*-SLP-1 does not show any membrane-dissolving activity, since it may indeed perform a scissor-like motion similar to that proposed for granulysin [14]. However, a noteworthy difference between *Na*-SLP-1 and *Ac*-SLP-1 in the third phase of the monolayer adsorption experiments is the slow but continuous decline in surface pressure with *Na*-SLP-1 binding after reaching the maximum value. Potentially, *Na*-SLP-1 may extract lipid molecules and then detach from the monolayer with a bound lipid molecule, but without compromising the integrity of the remaining monolayer.

The various dimer conformations seen in the crystal packing of *Na*-SLP-1 indicate that several oligomeric membrane-bound states of this protein may exist. Interestingly, the dimer with the largest interaction interface has an extended basic surface patch and would constitute an ideal conformation for initial membrane contact (Figure 3). Other dimer conformations, where the basic surface patch is located on different faces of the dimeric species, may be able to simultaneously contact adjacent membrane surfaces and thus mediate membrane aggregation.

### Proposed membrane binding mechanisms for *Ac*-SLP-1

Size exclusion chromatography and light scattering confirms that *Ac*-SLP-1 exists as a dimer in solution. The crystallographic dimer observed in the hexagonal crystal structure is very likely to represent the dimeric species in solution due to its large buried surface area. A significant area of this interface is hydrophobic and dimer formation leads to a favourable covering of this hydrophobic patch. Four inter-molecular hydrogen bonds further contribute to the stability of this dimer.

This dimeric *Ac*-SLP-1 species has implications for the membrane interactions of this SAPLIP (Figure 5) and is in agreement with the observations made in the monolayer adsorption experiments. When injecting the protein into the sub-phase of the membrane monolayer in the film balance experiments, the protein exists as a hydrophilic dimer that eventually gets into contact with the membrane. A transient interaction with the membrane could then trigger disassembly of the dimer, and the liberated monomers roll themselves towards the membrane in such an orientation that the flat hydrophobic surface (helix α3) faces the membrane. The trigger event that leads to disassembly of the membrane-associated dimers may come from the tryptophan residue at position 48, which sits in the link area between helices α2 and α3. The side chain of this residue is buried in a cleft, but can be rotated out without requiring any further structural rearrangements. This tryptophan is conserved in granulysin (Trp-41), and has been suggested to insert into the membrane during the initiation of membrane disruption caused by the protein [14]. For comparison, *Na*-SLP-1 has a tyrosine residue in this position, which is less likely to perform the same activity. NK-lysin seems to employ a tryptophan residue in a different location (Trp-58 in α4) to aid in the membrane anchoring process [32].

Once anchored at the membrane, *Ac*-SLP-1 is likely to establish electrostatic contacts between the isolated clusters of basic residues located on α1 and α3 and the polar interface of the membrane. A driving force to submerge helix α3 into the membrane layer may be provided by Lys-36 on helix α2 establishing a further electrostatic contact. Lys-36 may be located just below the membrane surface, and a direct interaction with the phospholipid head groups or the membrane interfacial region would push the protein into the membrane layer.

At acidic pH, acidic residues (Glu-55, Glu-63) located just underneath the hydrophobic surface that is suggested to be embedded in the membrane (see Figure 5) will become protonated and thus no longer carry a negative charge. The loss of charge in this area would support the embedding process as can clearly be seen by the larger surface pressure changes under acidic conditions (Figure 4).

The proposed membrane-bound state of *Ac*-SLP-1 would leave a large entry/exit cavity lined by residues from helices α1, α2 and α3 accessible to shuttle molecules between the membrane surface and the hydrophilic medium below.

### Functional role and comparison with other SAPLIPs

The process by which *N. americanus* lyses erythrocytes to liberate haemoglobin is not understood. Attempts to characterise haemolytic proteins from homogenates of *N. americanus* have been unsuccessful. Haemolytic proteins have been identified in two parasitic helminths, *C. sinensis* and *F. hepatica*, respectively. Both of these parasites are liver flukes (phylum Platyhelminthes) and therefore belong to a distinct phylum to the hookworms (Nematoda). In both of these liver flukes, the haemolytic proteins were members of the SAPLIP family, and given the conservation in digestive proteases between parasitic platyhelminths [33] and nematodes [34], we hypothesised that hookworm SAPLIPs may be haemolytic proteins.

The amoebapores appear to be the only pore-forming proteins in *E. histolytica* [13], yet at least a further 12 genes containing SAPLIP domains have been identified [35]. A comparison of the two hookworm SAPLIPs with other SAPLIPs highlights the diverse functionalities of proteins that contain a Sap domain. We could not demonstrate haemolysis and antibactericidal properties for either *Ac*-SLP-1 or *Na*-SLP-1 and it may be that one or more of the estimated six SAPLIPs currently thought to be encoded in the *A. caninum* genome [26], and the similar number inferred to be expressed by *N. americanus*, may show these functions. Alternatively, the lytic activity of the two hookworm SLP-1 proteins may arise only when they are part of a properly assembled protein complex.

The genome of the free-living nematode *Caenorhabditis elegans* also contains multiple SAPLIP sequences. Since this nematode is not a blood feeder, these proteins clearly have non-haemolytic functions in this free-living nematode, most of which are yet to be defined. One *C. elegans* SAPLIP, caenopore 5 (SPP-5), is a pore forming molecule with structural and sequence similarities to amoebapores [36]. Liposome depolarization assays indicate that this molecule effectively lyses bacterial membranes. RNAi suppression of SPP-5 results in a number of phenotypes associated with poor feeding activity and increased survival of *Escherischia coli*, the food source of *C. elegans*, in the worm intestine [36]. SPP-5 has weak similarity with *Ac*-SLP-1 (25% amino acid sequence identity). Similarly, *C. elegans* SPP-1, a SAPLIP expressed in the intestine possesses antibacterial activity and its expression is regulated by DAF-2/insulin receptor pathways signaling and by the presence of bacterial pathogens in the intestine [37].

Despite having different molecular mechanisms for membrane interactions, a similar membrane-bound state for both hookworm SAPLIPs can be suggested based on the data presented in the current study. Both proteins may be inserted partially into the membrane, and we suggested the surfaces likely to be embedded.

*Ac*-SLP-1 features a large entry/exit cavity that may connect the interface between the protein and the membrane with the surrounding solvent phase. Lipid molecules could be received there and handed over to another protein yet to be identified. The function of *Na*-SLP-1, in contrast, may be similar to granulysin since both seem to share a scissor-like motion of the two “jaws”. A potential mechanism could employ *Na*-SLP-1 as a lipid storage or carrier protein that works collaboratively with a lytic protein. The fold of the Sap domain has previously been suggested to provide a versatile scaffold for protein-protein interactions [38]. The crystal structure of granulysin revealed extensive protein-protein interactions in two dimensions, suggesting a model of a granulysin 2d-layer covering the membrane surface [14]. In this context, the observation of several dimers of *Na*-SLP-1 in the hexagonal crystal form is of great importance. This indicates that the protein is capable of extended protein-protein interactions, albeit an ultrastructural prediction cannot be made based on the currently available data.

## Materials and Methods

### Bioinformatics

Databases were constructed from GenBank cDNA entries as of August 2008, and searched for the distinctive cysteine spacing pattern of the Sap domain. Two patterns were used: one that allows for extra cysteine residues between the family-defining cysteine residues (C[∧C]{2}C[∧C]{23,28}C[∧C]{8,13}C[∧C]{23,26}C[∧C]{5,7}C), and one that only allows for the canonical cysteine residues (C.{2}C.{23,28}C.{8,13}C.{23,26}C.{5,7}C).

### Cloning, expression, purification and identification of proteins

The cDNA sequences of Na-slp-1 and *Ac-slp-1* were amplified from *N. americanus* whole worm cDNA and *A. caninum* gut cDNA [25] using established PCR protocols. The amplified products were ligated into the pPICZαA vector (Invitrogen) using the Xho1 and Not1 restriction sites.

*P. pastoris* colonies resistant to 500 µg/ml of zeocin were selected for protein expression in a 1 L biomass culture as per the manufacturer's instructions and with methanol added to a final volume of 0.5% every 24 hours for five days.

Secreted proteins were subjected to cation exchange chromatography (HiTrapp SP, 5 ml GE Healthcare), and the pooled eluates were dialysed into 100 mM sodium citrate pH 5 and concentrated using Amicon Ultra centrifugal filter devices with a pore size of 3 kDa (Millipore). The final concentration of the protein was determined densitometrically, using SDS-PAGE gels with BSA standards. The identity of purified recombinant *Na*-SLP-1 and *Ac*-SLP-1 was confirmed by MS fingerprinting. ESI-MS was used to further confirm the identity of the purified proteins. Several C-terminal truncated variants were observed, including *Na*-SLP-1(1–96), *Na*-SLP-1(1–101), *Na*-SLP-1(1–102), and *Na*-SLP-1(1–105) (data not shown). The MS data showed that neither protein is post-translationally modified.

### Size exclusion chromatography with light scattering (SEC-MALS)

Purified recombinant proteins were subjected to size exclusion chromatography with multi-angle light scattering detection (SEC-MALS) using a Superose-12 column. The SEC-MALS combination consisted of a Bio-Rad DuoFlow HPLC coupled to a Wyatt miniDawn TREOS light scattering detector and a Shimadzu RID-10A refractive index detector. The samples were injected at a concentration of 5–10 mg mL^−1^, and the buffer conditions were 100 mM NaCl, 20 mM HEPES (pH 7.5) or 100 mM Na-citrate (pH 5.0). MALS analysis was carried out using the Wyatt ASTRA software; peak analysis and integration was carried out with the program SDAR [39].

### Protein crystallisation

Initial crystallisation screens were carried out at 16°C in sitting drops in 96-well MRC crystallisation plates (Molecular Dimensions Ltd, Suffolk, England) and our large in-house factorial collection (more than 1000 pre-formulated conditions).

For *Na*-SLP-1, bipyramidal crystals (ca 0.1 mm on the vertical axis) were grown in 24-well plates with hanging drop setup (2+2 µl), using a protein stock solution of 10 mg/ml and a reservoir solution of 0.2 M NaCl, 20% PEG 6000, 0.1 M HEPES, pH = 7. Crystals appeared after 1–2 weeks.

In case of *Ac*-SLP-1, the best diffracting crystals were obtained from 1.35 M sodium citrate, 0.1 M HEPES, pH 7.8, in sitting drop plates, and had the shape of rectangular prisms.

### X-ray diffraction data collection and structure determination

Crystals were prepared for X-ray diffraction by brief immersion in mother liquor containing 25% glycerol and flash freezing in liquid nitrogen. X-ray diffraction data was collected at the in-house diffractometer (Rigaku MicroMax007-HF; R-Axis IV++ detector; Rigaku X-stream cryo equipment), and at beam lines MX1 and MX2 of the Australian Synchrotron under cryogenic conditions (T = 100 K). Datasets were indexed with XDS [40] and Mosflm [41], and scaling, truncation and analysis was performed with programs from the CCP4 suite [42].

In the case of *Na*-SLP-1, eight Pt sites were located and subjected to heavy atom refinement, as well as subsequent density modification and solvent flattening, yielding a phasing power of 0.514/ 0.611/0.916 (iso acentric, iso centric, ano acentric) and a mean figure of merit of 0.158/0.261 (centric/acentric). For *Ac*-SLP-1, two Pt sites were located with a phasing power of 2.11/1.14/1.32 (iso acentric, iso centric, ano acentric) and a mean figure of merit of 0.790/0.702 (centric/acentric). For data collection and refinement statistics see Tables 2 and 3. The geometry of the final models was scrutinised with PROCHECK [43] and the following Ramachandran statistics were obtained: *Na*-SLP-1 molecule A 96.1/2.6/1.3/0, *Na*-SLP-1 molecule B 89.9/8.8/1.3/0, *Ac*-SLP-1 86.1/11.1/2.8/0 (% allowed/additionally allowed/generously allowed/disallowed). Interface analysis was done with PISA [44]. For further details, please see Materials and Methods S1.

**Table 2:** Data collection and refinement statistics for *Na*-SLP-1.

	Native	*Na*-SLP-1:K_2_PtI_6_	Native (high res.)
**Data collection**			
Space group	P6_5_22	P6_5_22	P6_5_22
Cell dimensions			
*a*, *b*, *c* (Å)	85.1, 85.1, 117.8	85.1, 85.1, 119.1	85.4, 85.4, 113.5
α, β, γ (°)	90, 90, 120	90, 90, 120	90, 90, 120
Wavelength (Å)	0.95379	0.99195	0.94723
Resolution (Å)	3.0	3.2	2.7
*R*_sym_	0.093 (0.184)	0.009 (0.427)	0.101 (0.515)
*I*/σ*I*	4.4 (2.4)	7.6 (1.8)	7.4 (1.5)
Reflections with I/σI>2 (%)	100	83	88
Completeness (%)	100 (100)	100 (100)	99.9 (100)
Redundancy	40.0 (42.0)	40.9 (42.5)	41.7 (43)
**Refinement**			
Resolution (Å)			2.7
No. reflections: work/test			6854/337
*R*_work_/*R*_free_			0.185 (0.211)/0.245 (0.271)
No. atoms			
Protein			1400
Water			41
*B*-factors			
Protein			40.5
Water			39.3
R.m.s deviations			
Bond lengths (Å)			0.008
Bond angles (°)			1.147
bonded B-factors (Å^2^)			4.41

Datasets were obtained from one crystal each. Values in parentheses are for highest-resolution shell.

**Table 3:** Data collection and refinement statistics for *Ac*-SLP-1.

	Native	*Ac*-SLP-1:PtO_2_
**Data collection**		
Space group	P6_2_22	P6_2_22
Cell dimensions		
*a*, *b*, *c* (Å)	72.1, 72.1, 90.9	71.5, 71.5, 90.7
α, , β, , γ (°)	90, 90, 120	90, 90, 120
Wavelength (Å)	0.9536	1.5418
Resolution (Å)	2.3	3.3
*R*_sym_	0.079 (0.461)	0.092 (0.282)
*I*/σ*I*	7.3 (1.6)	8.2 (2.8)
Reflections with I/σI>2 (%)	86	100
Completeness (%)	100 (100)	100 (100)
Redundancy	18.7 (14.9)	18.9 (19.2)
**Refinement**		
Resolution (Å)	2.3	
No. reflections: work/test	6313/313	
*R*_work_/*R*_free_	0.225 (0.271)/0.252 (0.340)	
No. atoms		
Protein	606	
Ligand/ion	28	
Water	32	
*B*-factors		
Protein	48.9	
Ligand/ion	50.9	
Water	49.3	
R.m.s deviations		
Bond lengths (Å)	0.008	
Bond angles (°)	1.183	
bonded B-factors (Å^2^)	6.92	

Datasets were obtained from one crystal each. Values in parentheses are for highest-resolution shell.

### Liposome co-pelleting and stability assays

To assess membrane binding of hookworm SAPLIPs as well as their ability to destabilise liposomal membranes under a variety of different conditions, liposome co-pelleting and stability assays were conducted following published protocols [45]. For the co-pelleting assay, equal sized aliquots of samples and control were subjected to SDS-PAGE, gels were stained with Coomassie and analysed densitometrically using ImageJ [46]. An aggregation control assay was run using the same sample compositions, but replacing the liposome suspension by an equal volume of buffer. All experiments were carried out three times independently. For the stability assay, the water-soluble fluorescence dye carboxyfluoresceine diacetate (CF) is enclosed in liposomes at self-quenching concentrations and fluorescence emission spectra (λ_exc_ = 480 nm) were recorded from 490 to 600 nm using a Cary Eclipse Fluorescence Spectrophotometer. Protein at a final concentration of c = 1–5 mM was added only after monitoring the baseline fluorescence for 5 min. Complete disruption of the liposomes was achieved by adding 0.1% Triton X-100 (final concentration) which yielded the maximum possible fluorescence for each sample. Control experiments in the absence of protein were conducted by adding comparable amounts of protein buffer.

### Monolayer adsorption measurements

Measurement of protein adsorption to phospholipid monolayers was carried out using a computer-controlled Langmuir surface film balance (NIMA model 301A) at 20°C following a protocol published earlier [47]. The protein was injected into the sub-phase at a final concentration of 0.2–0.3 µM (corresponding to a relative amount of protein per lipid surface of 0.5–0.9 nmol cm^−2^) using a Hamilton syringe extending beneath the barrier, and the surface pressure π was recorded as a function of time. Adsorption data were analysed with the software SDAR from the PCSB program collection [39].

### Accession numbers

Atomic coordinates and structure factors have been deposited with the Protein Data Bank, accession numbers 3s63 and 3s64.

## Supporting Information

Materials and Methods S1 **Contains additional experimental procedures (antibody production, structure determination, void volume calculation, haemolysis assay, bactericidal assay).**
(DOC)

Figure S1 **Shows the atomic displacement parameter (B-factor) mapping on the structures of** ***Ac*** **-SLP-1 and** ***Na*** **-SLP-1.** Cartoon representations of the crystal structures of *Ac*-SLP-1 (**top**) and *Na*-SLP-1 (**bottom**) are coloured according to the B-factors of individual residues. The colour spectrum used to represent the B-factors is shown in the middle of the figure; low B-factors are represented by the colour blue, while high B-factors are represented by the colour red.
(TIF)
