# Supplementary material for: Insights into the Membrane Interactions of the Saposin-Like Proteins Na-SLP-1 and Ac-SLP-1 from Human and Dog Hookworm
Source: PLoS One. 2011 Oct 3;6(10):e25369. doi: 10.1371/journal.pone.0025369 (PMC3184995; doi:10.1371/journal.pone.0025369)
Supplement: Materials and Methods S1 — Contains additional experimental procedures (antibody production, structure determination, void volume calculation, haemolysis assay, bactericidal assay). (DOC) [file pone.0025369.s001.doc]

**Materials and Methods S1**

**Antibody production**

The coding sequence of the mature *Ac*-SLP-1 and *Na*-SLP-1 proteins (after removal of the predicted signal peptide) were cloned into the pET41a vector (Novagen) and expressed in BL21(DE3) cells as insoluble inclusion bodies with an approximate mass of 10 and 17 kDa as observed on SDS-PAGE. The proteins were purified via nickel affinity chromatography (Ni-NTA resin, QIAGEN) using denaturing conditions (6 M urea) according to the manufacturer’s instructions. The denatured recombinant protein was used to raise polyclonal antibodies in female BALB/c mice. Four mice were injected with 50 µg of protein emulsified in Freund’s Complete Adjuvant for the first immunisation and Freund’s Incomplete Adjuvant for two boosts occurring three weeks apart. Mice were euthanized two weeks after the last boost and whole blood was obtained via cardiac puncture. Serum was obtained by centrifugation at 5000 *g* for 5 min and stored at -20°C until required. Cross-reactivity between *Na*-SLP-1, *Ac*-SLP-1 and *Ac*-SLP-2 [1] was checked via standard Western Blot methodology.

**Structure determination**

Initial attempts to solve the structure by molecular replacement using 21 search models constructed from seven different structures (PDB accession codes 1m12, 1of9, 1sn6, 2gtg, 2jsa, 2qyp, 2z9a) were unsuccessful.

The structures were determined using single isomorphous replacement with anomalous diffraction (SIRAS) with data from a Pt derivative and a native crystal each. In total, for *Na*-SLP-1 four datasets of native crystals, as well as 29 datasets of crystals derivatised with 12 heavy atom compounds were collected. With *Ac*-SLP-1 we analysed 4 native and 12 derivatised crystals with 7 heavy atom compounds.

Eight (*Na*-SLP-1) and two (*Ac*-SLP-1) heavy atom sites were located with SHELX [2] as implemented in CCP4, and subjected to heavy atom refinement, as well as subsequent density modification and solvent flattening with autoSHARP [3]. Using the initial phases, the programs Buccaneer [4] and HELICAP [5] (*Na*-SLP-1) were able to build Ca traces of 2 helices of one molecule. Informed by the topology of the saposin fold, 5 helices were then placed manually in the density with Coot [6]. A similar strategy worked for *Ac*-SLP-1 using Auto-Rickshaw [7]. For *Na*-SLP-1, the location of the second molecule in the asymmetric unit could be identified using the skeleton of the first molecule as a rigid body. For further model building, composite omit maps were calculated with CNS [8], and multiple rounds of manual model building with O [9] were interspersed with computational refinement with (rigid body refinement, positional refinement, NCS restraints in case of *Na*-SLP-1) using Phenix [10]. Once 75% of the backbone were traced, the models were placed in the datasets of native crystals with highest resolution by a molecular replacement calculation. Final structure refinement was carried out using these datasets. Solvent and buffer molecules were placed manually, and individual B-factors could be refined.

In *Na*-SLP-1, the C-terminal extension comprising about 27 residues were not visible in the electron density. The mass of the protein used for crystallisation was thus verified again using electrospray ionisation mass spectrometry. Several C-terminal truncated variants were observed, including *Na*-SLP-1(1-96), *Na*-SLP-1(1-101), *Na*-SLP-1(1-102), and *Na*-SLP-1(1-105) (data not shown).

The crystal structure of *Ac*-SLP-1 comprises residues 7-87 (side chains modelled as Ala: Asp-30, Gln-33, Lys-36) and has rather high thermal displacement factors which may be due to the high solvent content of the crystals.

**Void volume calculation**

Cavities of molecular models were evaluated with the program VOIDOO [11]. The probe radius was set to 0.5 Å rather then the default value of 1.4 Å, in order to assess volumes between core side chains and not space compartments that may be available to a ligand [12].

**Haemolysis assay**

Haemolysis assays were performed using the methodology of Don and colleagues [13]. In brief, 1% v/v of human red blood cells were added to various amounts of protein (50 ng to 4 µg final masses) in the presence or absence of metal ions (ZnCl_2_, MgCl_2_ and CaCl_2_) in 100 mM sodium citrate buffer pH 5 or pH 7. The samples were incubated at 37°C for up to 90 min. Haemolysis was assessed by subjecting the supernatant to spectrometric measurement of absorption at 540 nm using positive (deionised water) and negative (buffer only) controls as 100% lysis and 0% lysis, respectively. Control proteins were Scabies Mite Inactive Protease Paralogues (SMIPPs) and *S. mansoni* Tetraspanin 2 (*Sm*-TSP-2). These controls were chosen, because they were obtained as recombinant proteins from yeast expression and had specific antisera raised against bacterially expressed proteins in a similar way as described above for the hookworm SAPLIPs.

**Bactericidal assay**

10 mL of LB broth were inoculated with *E. coli* TOP10 cells and grown overnight at 37°C with shaking. 500 µL of the culture were then spread onto YB agar plates and dried. Small discs of Whatman paper were soaked in *Na*-SLP-1 protein at differing concentrations just prior to placement on the agar plates. To calculate zones of inhibition, negative controls (LB only as well as sodium citrate only) and a positive control (kanamycin) were used. Plates were incubated at 37°C overnight.

**Supplemental References**

1. Don TA, Oksov Y, Lustigman S, Loukas A (2007) Saposin-like proteins from the intestine of the blood-feeding hookworm, *Ancylostoma caninum*. Parasitology 134: 427-436.

2. Sheldrick G (2008) A short history of SHELX. Acta Cryst. A 64:112-122.

3. Vonrhein C, Blanc E, Roversi P, Bricogne G (2007) Automated structure solution with autoSHARP. Methods Mol. Biol. 364: 215-230.

4. Cowtan K (2008) Fitting molecular fragments into electron density. Acta Crystallogr. D 64: 83-89.

5. Morris R, Zwart P, Cohen S, Fernandez F, Kakaris M, et al. (2004) Breaking good resolutions with ARP/wARP. J. Synchrotron Rad. 11: 56-59.

6. Emsley P, Cowton K (2004) Coot: Model-Building Tools for Molecular Graphics. Acta Crystallogr. D60: 2126-2132.

7. Panjikar S, Parthasarathy V, Lamzin V, Weiss M, Tucker P (2005) Auto-Rickshaw: an automated crystal structure determination platform as an efficient tool for the validation of an X-ray diffraction experiment. Acta Crystallogr. D 61: 449-457.

8. Brünger A, Adams P, Clore G, Delano W, Gros P, et al. (1998) Crystallography and NMR system (CNS): A new software system for macromolecular structure determination. Acta Crystallogr D54: 905-921.

9. Jones TA, Zou JY, Cowan S, Kjeldgaard M (1991) Improved methods for building protein models in electron density maps and location of errors in these models. Acta Crystallogr. A 47: 110-119.

10. Adams PD, Afonine PV, Bunkóczi G, Chen VB, Davis. I. W., et al. (2010) PHENIX: a comprehensive Python-based system for macromolecular structure solution. Acta Crystallogr. D66: 213-221.

11. Kleywegt G, Jones T (1994) Detection, delineation, measurement and display of cavities in macromolecular structures. Acta Crystallogr. D 50: 178-185.

12. Anderson DH, Sawaya MR, Cascio D, Ernst W, Modlin R, et al. (2003) Granulysin crystal structure and a structure-derived lytic mechanism. J Mol Biol 325: 355-365.

13. Don TA, Jones MK, Smyth D, O'Donoghue P, Hotez P, et al. (2004) A pore-forming haemolysin from the hookworm, *Ancylostoma caninum*. Int. J.Parasitol. 34: 1029–1035.
