# Supplementary figures and images for: Insights into the Membrane Interactions of the Saposin-Like Proteins Na-SLP-1 and Ac-SLP-1 from Human and Dog Hookworm
Source: PLoS One. 2011 Oct 3;6(10):e25369. doi: 10.1371/journal.pone.0025369 (PMC3184995; doi:10.1371/journal.pone.0025369)

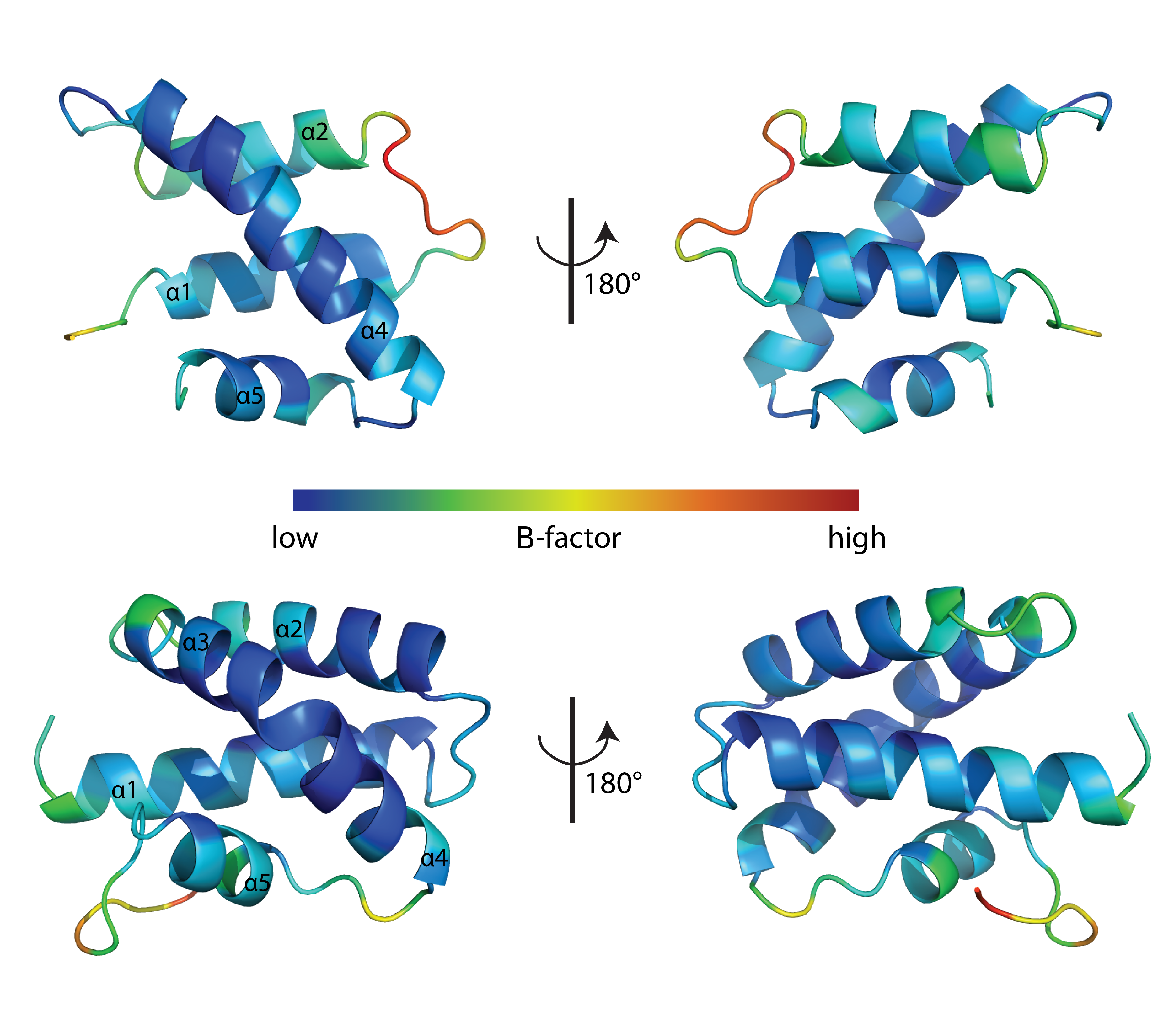

Supplement: Figure S1 — Shows the atomic displacement parameter (B-factor) mapping on the structures of Ac -SLP-1 and Na -SLP-1. Cartoon representations of the crystal structures of Ac-SLP-1 (top) and Na-SLP-1 (bottom) are coloured according to the B-factors of individual residues. The colour spectrum used to represent the B-factors is shown in the middle of the figure; low B-factors are represented by the colour blue, while high B-factors are represented by the colour red. (TIF) [file pone.0025369.s002.tif]
